# Single-crystal structure determination of two new ternary bis­muthides: Rh_6_Mn_5_Bi_18_ and RhMnBi_3_

**DOI:** 10.1107/S2053229618009087

**Published:** 2018-06-28

**Authors:** Peter Kainzbauer, Klaus W. Richter, Herta Silvia Effenberger, Martin C. J. Marker, Herbert Ipser

**Affiliations:** a Department of Inorganic Chemistry – Functional Materials, University of Vienna, Faculty of Chemistry, Althanstrasse 14, Vienna 1090, Austria; b Institute of Mineralogy and Crystallography, University of Vienna, Althanstrasse 14, Vienna 1090, Austria

**Keywords:** Rh_6_Mn_5_Bi_18_, RhMnBi_3_, crystal structure, Rh-Mn-Bi phase diagram, ternary bis­muthide, intermetallic, pnictide

## Abstract

A study of the ternary Rh–Mn–Bi phase diagram revealed the existence of two new ternary bis­muthides, *viz.* hexa­rhodium penta­manganese octa­deca­bis­muthide (Rh_6_Mn_5_Bi_18_) and rhodium manganese tribismuthide (RhMnBi_3_). Their crystal structures represent new structure types. Rh_6_Mn_5_Bi_18_, with a Wyckoff sequence *a* *f*2 *g*2* i*5, crystallizes in the tetra­gonal system (space group *P*4_2_/*mnm*; Pearson symbol *tP*58), and RhMnBi_3_, with a Wyckoff sequence *a c g i q*, crystallizes in the ortho­rhom­bic system (*Cmmm*; *oS*20). In the Rh_6_Mn_5_Bi_18_ structure, the transition metal atoms are linked into ribbon-like structural units aligned along the [001] direction, whereas planar sheets are formed in RhMnBi_3_. In both crystal structures, the units formed by the transition metal atoms are enveloped by Bi atoms, which themselves form a loosely bound network. The linkage results in a layer structure for RhMnBi_3_, while in the case of Rh_6_Mn_5_Bi_18_, a three-dimensional network is formed; the latter, however, contains several areas where Bi⋯Bi distances suggest van der Waals inter­actions. Both phases under discussion have analogous structural motifs.

## Abstract 2

A study of the ternary Rh–Mn–Bi phase diagram revealed the existence of two new ternary bis­muthides, *viz.* hexa­rhodium penta­manganese octadecabismuthide (Rh_6_Mn_5_Bi_18_) and rhodium manganese tribismuthide (RhMnBi_3_). Their crystal structures represent new structure types.

## Introduction   

For decades, there has been an ongoing search for ferromagnetic materials free of rare earth elements. One promising candidate is the inter­metallic phase α-BiMn; unfortunately, it has not been possible to synthesize this phase as a single-phase bulk material in spite of intensive research (*e.g.* Liu *et al.*, 2004 ▸; Rama Rao *et al.*, 2013 ▸; Cui *et al.*, 2014 ▸; Chen *et al.*, 2015 ▸; Marker *et al.*, 2018 ▸). A possible approach to circumvent these problems was considered to be the addition of a third com­ponent, *e.g.* Rh, which forms an inter­metallic phase with Bi that is isotypic with α-BiMn (Ross & Hume-Rothery, 1962 ▸; Kainzbauer *et al.*, 2018 ▸).

Street *et al.* (1974 ▸) identified a ferromagnetic compound, *i.e.* Mn_5_Rh_2_Bi_4_ (cubic, *Fm* 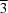 *m*), with a Curie temperature of 266 K. A similar observation was made by Taufour *et al.* (2015 ▸), who described the ferromagnetic compound Mn_1.05_Rh_0.02_Bi, with a Curie temperature below 416 K. Furthermore, Suits (1975 ▸) discovered ferromagnetism in Bi-substituted RhMn with the composition RhMn_0.8_Bi_0.2_. Based on these observations, a systematic study of the ternary Rh–Mn–Bi system at different temperatures was considered of inter­est, with the focus on finding additional inter­metallic phases which might possibly exhibit ferromagnetism. The synthesized samples were checked by powder X-ray diffraction (PXRD) investigations. As a result of this ongoing research, the phases hexa­rhodium penta­manganese octa­deca­bis­muthide (Rh_6_Mn_5_Bi_18_) and rhodium manganese tribismuthide (RhMnBi_3_) were detected; admittedly, they are not ferromagnetic.

A literature survey of the ternary Rh–*M*–Bi systems (*M* = 3*d* transition metal) shows that they are relatively unexplored. Except for the aforementioned phases, only a handful of compounds are known. Examples are RhNiBi_2_ (Zhuravlev *et al.*, 1962 ▸) and RhNiBi_6_ (Fjellvåg & Furuseth, 1987 ▸). It may be of particular inter­est that Rh_6_Mn_5_Bi_18_ is probably one of the first reported ternary manganese pnictide phases, with a network formed by Bi atoms where alkaline or rare earth metal elements are absent. Further examples are known to crystallize in the cubic structure type Cu_4_Mn_3_Bi_4_ (Street *et al.*, 1974 ▸; Szytula *et al.*, 1981 ▸).

## Experimental   

### Synthesis and crystallization   

Bulk samples were prepared from pure element pieces of Bi (99.999%, ASARCO, New Jersey, USA) and Mn (99.95%, Alfa Aesar, Johnson Matthey Chemicals, Karlsruhe, Germany), and from Rh powder (99.95% ÖGUSSA, Austria). Except for Rh, the metals were pulverized manually and sieved (grain size <0.09 mm). For the Rh_6_Mn_5_Bi_18_ phase, 76.40 mg Rh, 33.81 mg Mn and 389.26 mg Bi in powder form were mixed, and for RhMnBi_3_, the amounts were 95.14 mg Rh, 67.50 mg Mn and 838.14 mg Bi; in both cases, the powder mixtures were pressed into pellets in a 5 mm pressing cylinder under a load of 20–25 kN. The bulk samples for the Rh_6_Mn_5_Bi_18_ phase were sealed in an evacuated silica-glass tube and melted over an oxyhydrogen flame under shaking, with optical control of the melting process. For the alloying process, the samples were heated quickly to 1373 K, cooled over a period of 5 d to 613 K and annealed at this temperature for two weeks. The bulk samples for the RhMnBi_3_ phase were prepared as sinter pellets. The pellet was sealed in an evacuated silica-glass tube with a small alumina plate at the bottom and covered with an inverted closed silica-glass tube to reduce the gas volume (annealing time of four months). After the annealing process at 613 K in a muffle furnace (Nabertherm, Germany, temperature accuracy ±5 K), all samples were quenched in cold water.

Small single crystals of Rh_6_Mn_5_Bi_18_ and RhMnBi_3_ were obtained in several inhomogeneous bulk samples. The target compounds had a metallic luster and were selected manually using an optical stereomicroscope. Adherent bis­muth was removed with a scalpel. The entire preparation process was performed in an Ar-filled glove-box (Labmaster SP MBraun, H_2_O and O_2_ levels below 0.1 ppm). Differential thermal analysis was performed on a DSC 404F1 Pegasus (Netzsch, Selb, Germany) and showed that the Rh_6_Mn_5_Bi_18_ compound is stable up to 730 K. Phase identification was performed under ambient conditions by PXRD on a Bruker D8 Advance diffractometer in Bragg–Brentano pseudo-focusing geometry, using Cu *K*α radiation and a LynxEye^®^ one-dimensional silicon strip detector. Energy-dispersive X-ray spectroscopy analyses on a scanning electron microscope (Zeiss Supra 55 VP) confirmed that the elemental compositions corresponded to those from the single-crystal X-ray structure determination. Morphologically, both new bis­muthides are acicular and flaky.

### Refinement   

Crystal data, data collection and structure refinement details are summarized in Table 1 ▸. A number of crystal chips were checked for their scattering behaviour and, in particular, to exclude admixtures as adherent bis­muth. Crystals of sufficient quality were used for collection of the intensity data in the full reciprocal sphere. To minimize absorption effects, the crystals were mounted approximately parallel to the φ axes with their longest extension. As the crystal structures are composed of structural units only bonded by weak Bi—Bi bonds, extensive cleavage of the crystals is evident. As a consequence of this behaviour, only a crystal of limited quality could be found for Rh_6_Mn_5_Bi_18_, even though a large number of crystals was checked by single-crystal X-ray diffraction; thus, the *R*_int_ value and, consequently, the structure refinements remained poor. Nevertheless, the structure type could be clearly established.

A careful inspection of the reciprocal space gave no evidence for any superstructure reflections; twinning was not recognized. As mixed occupation of individual atom positions was not evident and the anisotropic displacement parameters were not conspicuous, a violation of centrosymmetry can be excluded within the accuracy of the structure refinements. Due to the high mosaicity of both samples, their extinction is neg­ligible. Complex neutral atomic scattering functions were applied (Prince, 2006 ▸). The program *STRUCTURE TIDY* (Gelato & Parthé, 1987 ▸) was used to standardize all atomic coordinates.

## Results and discussion   

As mentioned above, only a few pnictides are known with Rh and a second 3*d* transition metal as constituents (Street *et al.*, 1974 ▸; Szytula *et al.*, 1981 ▸; Huang *et al.*, 2015 ▸). The title phases are probably also the only reported ternary bis­muthides containing a platinum group element and Mn, which adopt new structure types.

**Figure 1:**
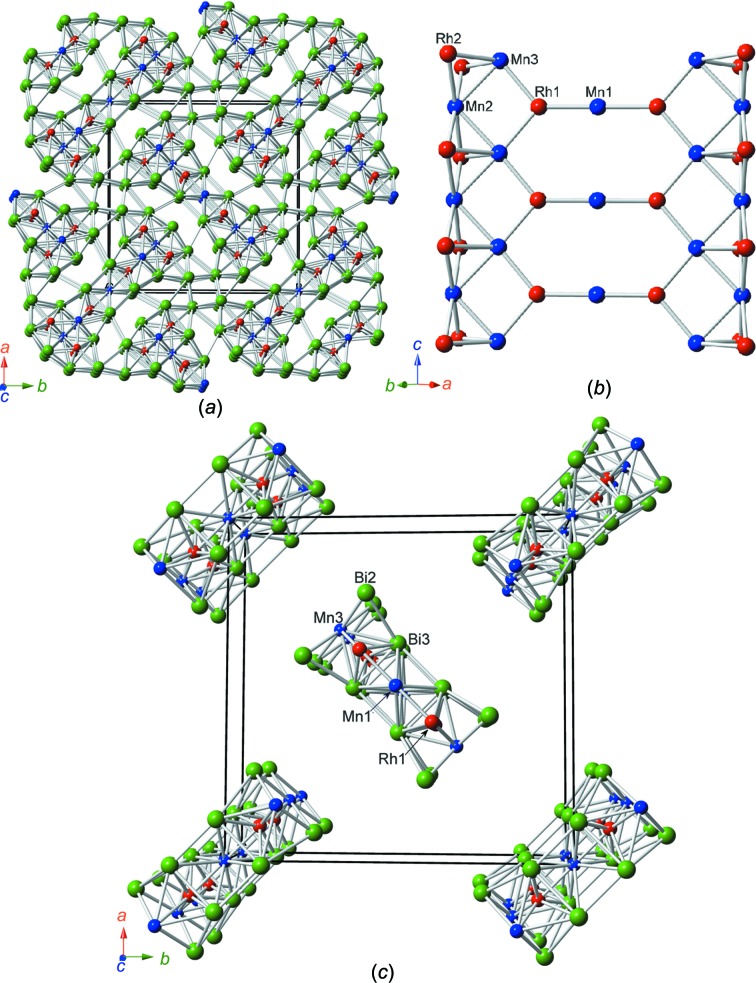
(*a*) The crystal structure of Rh_6_Mn_5_Bi_18_; the view is slightly inclined to the [001] direction. (*b*) The Rh_6_Mn_5_ ribbons (distances and angles are listed in Table 3 ▸). (*c*) A clinographic projection of the central parts of the ribbons (atoms Bi2, Bi3, Rh1, Mn1 and Mn3). Colour code: green represents Bi, blue Mn and red Rh atoms.

**Figure 2:**
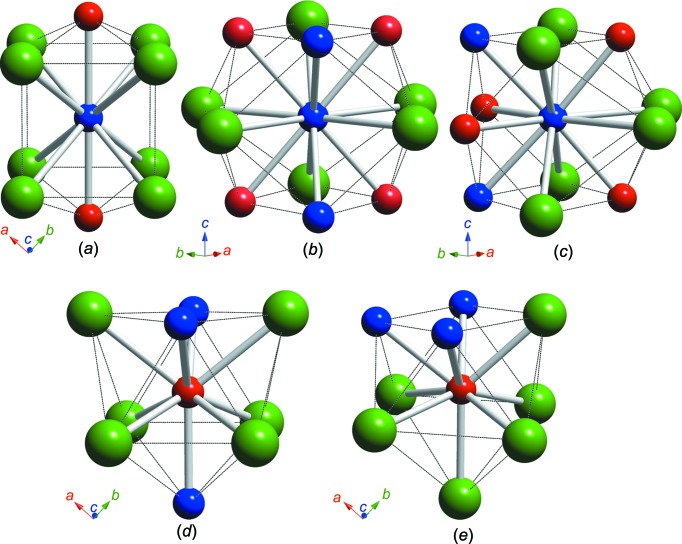
A schematic representation of the distinct atomic coordination spheres in the Rh_6_Mn_5_Bi_18_ structure. All neighbours within 3.2 Å are shown. (*a*) The bicapped square prism around the Mn1 atom [CN (coordination number) = 10]. (*b*) The distorted cubocta­hedron around the Mn2 atom (CN = 12). (*c*) The distorted cubocta­hedron around the Mn3 atom (CN = 12). (*d*) The tricapped trigonal prism (alternatively monocapped tetra­gonal anti­prism) around the Rh1 atom (CN = 9). (*e*) The capped square anti­prism (alternatively tricapped trigonal prism) around the Rh2 atom (CN = 9). The colour coding is as in Fig. 1 ▸.

Rh_6_Mn_5_Bi_18_ crystallizes in the tetra­gonal space group *P*4_2_/*mnm* (Pearson symbol *tP*58). The asymmetric unit contains ten atoms, which are listed together with their Wyckoff letters and site symmetries in Table 2 ▸. Fig. 1 ▸ shows the whole crystal structure and the main structural element of Rh_6_Mn_5_Bi_18_ formed by extensive linkage of the Mn and Rh atoms. It is characterized by double chains running parallel to [001], each with the formal composition Rh_3_Mn_2_. They are linked by an additional Mn1 atom to form ribbons with a linear Rh1—Mn1—Rh1 configuration. The central part of the chains consists of the atoms Rh2, Mn2 and Mn3, the Rh1 atom points towards the linking atom Mn1, and the Mn1 atom itself is surrounded in a bicapped square-prismatic coordination (CN = 10, position 2*a*) (see Fig. 2 ▸ *a* and Table 3 ▸). The ribbons are surrounded by Bi atoms, with Rh/Mn—Bi bond distances > 2.814 Å. All Bi atoms are exclusively bonded to one Rh_3_Mn_2_—Mn1—Rh_3_Mn_2_ ribbon. The Bi atoms themselves form an extended three-dimensional anionic network. The Bi—Bi bonds are longer than 3.316 Å; although Bi—Bi distances in the network were found up to 3.5808 (12) Å, which is slightly longer than the inter­layer Bi—Bi distance in native Bi under ambient conditions (3.529 Å; Donohue, 1974 ▸), bonding inter­actions are still implicated. In addition to the inter­atomic bonds, weak van der Waals Bi4⋯Bi4 [3.920 (2) Å] and Bi2⋯Bi5 [3.848 (1) Å] inter­actions contribute to the cohesion of the network. These longer distances are not shown in Fig. 1 ▸(*a*). The coordination spheres around all the transition-metal positions are depicted in Fig. 2 ▸.

A characteristic feature of the ribbons are eight-membered rings formed by two Mn1 and two Mn3 atoms, as well as four Rh1 atoms. In addition, four-membered rings are built by two Mn3, one Mn2 and one Rh1 atom. These two kinds of rings are planar by space-group symmetry. Only the Rh2 atoms are, respectively, above and below the layers; see Fig. 1 ▸(*b*). These structural units are the common structural motif of the two title compounds. However, tetra­gonal symmetry causes a herring-bone pattern of these one-dimensional structural units along [001] in Rh_6_Mn_5_Bi_18_, whereas they are linked into a two-dimensional arrangement in RhMnBi_3_ (see below).

**Figure 3:**
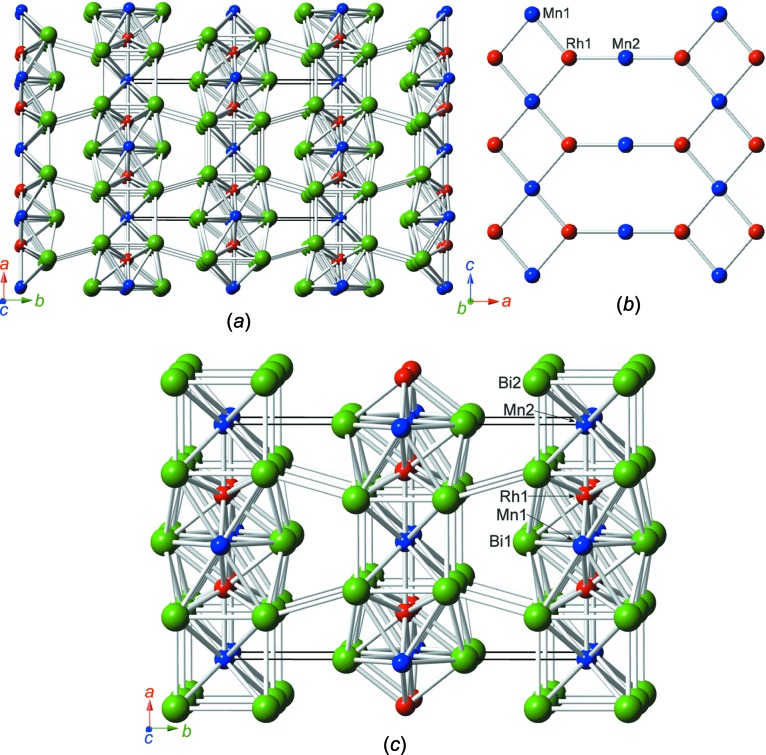
(*a*) The crystal structure of RhMnBi_3_, viewed approximately parallel to [001]. (*b*) The exactly planar Mn–Rh nets; the view is slightly inclined to the *b* direction (distances and angles are listed in Table 5 ▸). (*c*) A clinographic projection of the main structural elements parallel to [001]. The colour coding is as in Fig. 1 ▸.

**Figure 4:**
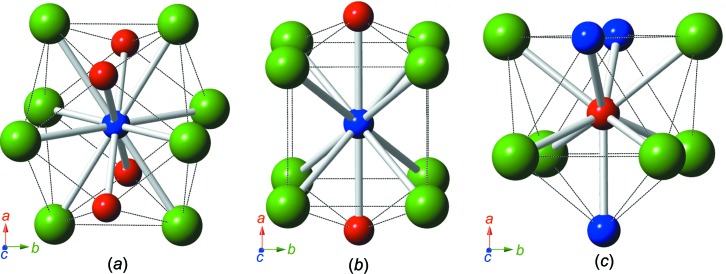
A schematic representation of different atomic coordination spheres in the RhMnBi_3_ structure, showing all neighbouring atoms up to a distance of 3.3 Å. (*a*) The monocapped square anti­prism around the Rh1 atom (CN = 9). (*b*) The distorted cubocta­hedron around the Mn1 atom (CN = 12). (*c*) The bicapped square prism around the Mn2 atom (CN = 10). The colour coding is as in Fig. 1 ▸.

RhMnBi_3_ crystallizes in the ortho­rhom­bic space group *Cmmm* (Pearson symbol *oS*20). Like Rh_6_Mn_5_Bi_18_, RhMnBi_3_ represents a new structure type and exhibits a layer structure consisting of planar Mn–Rh sheets parallel to (010) surrounded by Bi atoms, as presented in detail in Fig. 3 ▸. Fig. 3 ▸(*a*) shows the crystal structure along *c*, clearly indicating the layering. Bi—Bi bond distances between the layers are mainly in the range of van der Waals inter­actions, except for the Bi2⋯Bi2 distances of 3.590 (3) Å, which are slightly longer than the inter­layer distance in native Bi (3.529 Å), but are still assumed to exhibit weak bonding inter­actions. The planar nets formed by the transition metals shown in Fig. 3 ▸(*b*) consist of eight-membered rings of alternating Mn and Rh atoms, similar to the motif shown in Fig. 1 ▸(*b*). The coordination spheres around all the transition-metal positions are depicted in Fig. 4 ▸.

The asymmetric unit of the structure of RhMnBi_3_ itself contains five atoms, which are listed together with their Wyckoff letters and site symmetries in Table 4 ▸.

**Figure 5:**
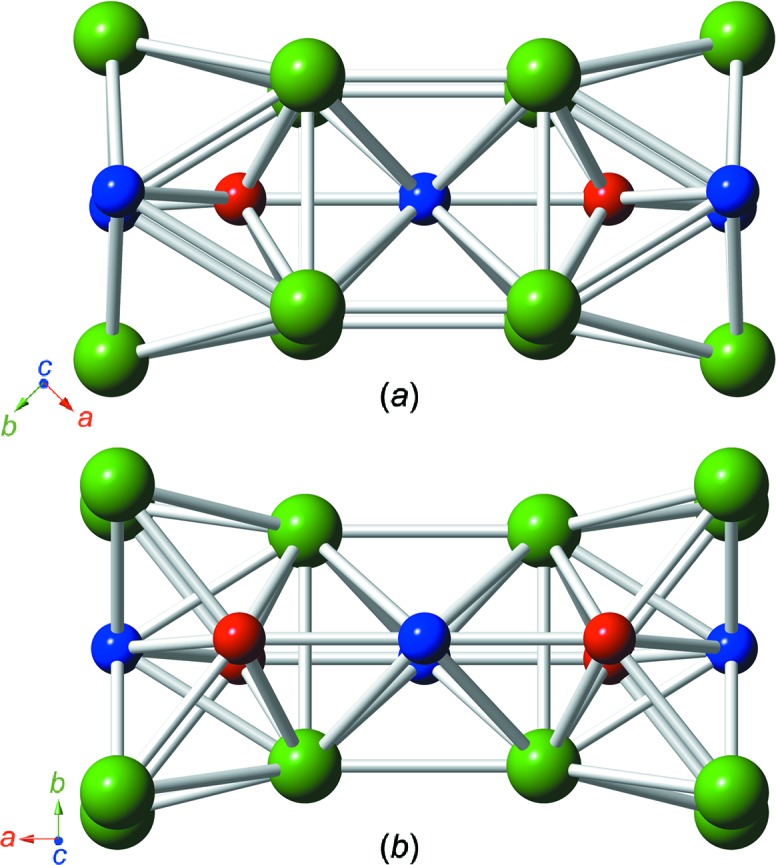
Comparison of the similar structural motif in the two new bis­muthides under discussion. (*a*) In Rh_6_Mn_5_Bi_18_, the motif consists of Bi2, Bi3, Mn2, Mn3 and Rh1 atoms. (*b*) In RhMnBi_3_, all distinct atom positions listed in Table 4 ▸ are included, and the motif is repeated infinitely forming two-dimensional layers (*cf.* Fig. 3 ▸). The colour coding is as in Fig. 1 ▸.

Finally, Fig. 5 ▸ illustrates clearly the relationship between the structures of Rh_6_Mn_5_Bi_18_ and RhMnBi_3_.

## Supplementary Material

Crystal structure: contains datablock(s) Rh6Mn5Bi18, RhMnBi3, global. DOI: 10.1107/S2053229618009087/sk3690sup1.cif

Structure factors: contains datablock(s) Rh6Mn5Bi18. DOI: 10.1107/S2053229618009087/sk3690Rh6Mn5Bi18sup3.hkl

Structure factors: contains datablock(s) RhMnBi3. DOI: 10.1107/S2053229618009087/sk3690RhMnBi3sup4.hkl

CCDC references: 1850893, 1850892

## Figures and Tables

**Table 1:** Experimental details

	**Rh_6_Mn_5_Bi_18_**	**RhMnBi_3_**
Crystal data		
Chemical formula	Rh_6_Mn_5_Bi_18_	RhMnBi_3_
*M*_r_	4653.80	784.79
Crystal system, space group	Tetragonal, *P*4_2_/*m* *n* *m*	Orthorhombic, *C* *m* *m* *m*
Temperature (K)	293	293
*a*, *b*, *c* (Å)	18.526 (3), 18.526 (3), 4.1722 (11)	8.885 (3), 13.696 (6), 4.1310 (12)
α, β, γ (°)	90, 90, 90	90, 90, 90
*V* (Å^3^)	1432.0 (6)	502.7 (3)
*Z*	2	4
Radiation type	Mo *K*α	Mo *K*α
μ (mm^−1^)	115.57	110.13
Crystal size (mm)	0.16 × 0.03 × 0.02	0.10 × 0.05 × 0.03

Data collection		
Diffractometer	Nonius KappaCCD	Nonius KappaCCD
Absorption correction	Multi-scan (*SCALEPACK*; Otwinowski & Minor, 1997 ▸)	Multi-scan (*SCALEPACK*; Otwinowski & Minor, 1997 ▸)
*T*_min_, *T*_max_	0.009, 0.011	0.003, 0.005
No. of measured, independent and observed [*I* > 2σ(*I*)] reflections	21380, 1784, 1297	3699, 667, 522
*R*_int_	0.169	0.141
(sin θ/λ)_max_ (Å^−1^)	0.803	0.806

Refinement		
*R*[*F* ^2^ > 2σ(*F* ^2^)], *wR*(*F* ^2^), *S*	0.044, 0.088, 1.05	0.094, 0.257, 1.19
No. of reflections	1784	667
No. of parameters	50	21
Δρ_max_, Δρ_min_ (e Å^−3^)	4.24, −3.66	11.68, −8.87

Computer programs: *COLLECT* (Hooft, 1999 ▸), *DENZO* and *SCALEPACK* (Otwinowski & Minor, 1997 ▸), *SHELXS97* (Sheldrick, 2008 ▸), *SHELXL2014* (Sheldrick, 2015 ▸) and *CrystalMaker* (CrystalMaker, 2009 ▸).

**Table 2:** Fractional atomic coordinates, Wyckoff letter and site symmetry of Rh_6_Mn_5_Bi_18_

	Wyckoff letter	Site symmetry	*x*	*y*	*z*
Bi1	4*g*	*m*.2*m*	0.17112 (3)	0.82888 (3)	0
Bi2	8*i*	*m*..	0.08340 (3)	0.25924 (3)	0
Bi3	8*i*	*m*..	0.37210 (3)	0.50115 (3)	0
Bi4	8*i*	*m*..	0.08560 (4)	0.56218 (4)	0
Bi5	8*i*	*m*..	0.20740 (4)	0.41210 (4)	0
Rh1	4*f*	*m*.2*m*	0.10090 (7)	0.10090 (7)	0
Rh2	8*i*	*m*..	0.18578 (7)	0.67667 (7)	0
Mn1	2*a*	*m.mm*	0	0	0
Mn2	4*f*	*m*.2*m*	0.24406 (14)	0.24406 (14)	0
Mn3	4*g*	*m*.2*m*	0.33193 (14)	0.66807 (14)	0

**Table 3:** Selected geometric parameters (Å, °) for Rh_6_Mn_5_Bi_18_

Rh1—Mn1	2.6435 (19)	Rh2—Mn2^ii^	2.7568 (10)
Rh1—Mn3^i^	2.729 (3)	Mn2—Mn3^iii^	2.884 (4)
Rh2—Mn3	2.712 (3)		
			
Mn1—Rh1—Mn3^iii^	130.15 (6)	Rh2^v^—Mn3—Mn2^ii^	58.93 (7)
Mn3^i^—Rh1—Mn3^iii^	99.69 (13)	Rh1^ii^—Mn3—Mn2^ii^	83.82 (7)
Rh1—Mn1—Rh1^iv^	180.00 (8)	Rh1^vi^—Mn3—Mn2^ii^	176.49 (13)
Rh2^v^—Mn3—Rh2	83.27 (12)	Rh1^vi^—Mn3—Mn2^vi^	83.82 (7)
Rh2^v^—Mn3—Rh1^ii^	118.81 (3)	Mn2^ii^—Mn3—Mn2^vi^	92.67 (15)
Rh1^ii^—Mn3—Rh1^vi^	99.69 (13)		

Symmetry codes: (i) 
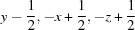
; (ii) 
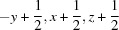
; (iii) 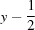, 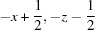; (iv) 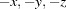; (v) 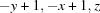; (vi) 
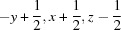
.

**Table 4:** Fractional atomic coordinates, Wyckoff letter and site symmetry of RhMnBi_3_

	Wyckoff letter	Site symmetry	*x*	*y*	*z*
Bi1	4*i*	*m*2*m*	0	0.33689 (14)	0
Bi2	8*q*	..*m*	0.19449 (14)	0.12399 (10)	½
Mn1	2*c*	*mmm*	½	0	½
Mn2	2*a*	*mmm*	0	0	0
Rh1	4*g*	2*mm*	0.3016 (4)	0	0

**Table 5:** Selected geometric parameters (Å, °) for RhMnBi_3_

Mn1—Rh1	2.715 (2)	Mn2—Rh1	2.680 (4)
			
Rh1^i^—Mn1—Rh1^ii^	99.05 (12)	Rh1^ii^—Mn1—Rh1	80.95 (12)
Rh1^i^—Mn1—Rh1^iii^	80.95 (12)	Mn2—Rh1—Mn1	130.48 (6)
Rh1^i^—Mn1—Rh1	180.0		

Symmetry codes: (i) 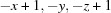; (ii) 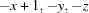; (iii) 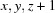.
